# Clinical success of anti-infective combination therapy compare to monotherapy in patients with carbapenem-resistant *Pseudomonas aeruginosa* infection: a 10-years retrospective study

**DOI:** 10.1186/s12879-024-09060-2

**Published:** 2024-02-23

**Authors:** Jialong Chen, Jing Lin, Jianzhen Weng, Yang Ju, Yanming Li

**Affiliations:** 1 Department of Pulmonary and Critical Care Medicine, Beijing Hospital,National Center of Gerontology, the Institute of Geriatric Medicine, Chinese Academy of Medical Sciences, Beijing, People’s Republic of China; 2 Graduate School, Peking Union Medical College, Chinese Academy of Medical Sciences, Beijing, China; 3 Department of Infectious Disease, Peking Union Medical College Hospital, Chinese Academy of Medical Sciences, Peking Union Medical College, Beijing, China

**Keywords:** Monotherapy, Combination therapy, Carbapenem-resistant *Pseudomonas aeruginosa*, Effectiveness

## Abstract

**Background:**

Carbapenem-resistant *Pseudomonas aeruginosa* (CRPA) infection has become a major public health concern. The recommendations for monotherapy and combination therapy in the current guidelines lack sufficient evidence to support them. The primary objective of this study is to determine the effectiveness of anti-Infective combination therapy compared to monotherapy in achieving clinical success in patients with CRPA infection and risk factors of clinical failure of monotherapy.

**Methods:**

A retrospective study from Medical Information Mart for Intensive Care IV (MIMIC-IV) was conducted. We included adults with infections caused by CRPA. The outcomes of this study were clinical success, complete clinical success, and 28-day all-cause mortality.

**Results:**

A total of 279 subjects were finally enrolled. The rate of clinical success for combination therapy was higher than that for monotherapy (73.1% versus 60.4%, *p*=0.028). Compared to clinical failure patients, patients in the clinical success group were more likely to die within 28 days after CRPA was found (48.3% versus 3.6%, *p*<0.001). In a multivariate logistic regression analysis, monotherapy was found to be significantly correlated with clinical success (OR, 0.559, 95% CI, 0.321-0.976; *p* = 0.041).

**Conclusion:**

Combination therapy is more effective for CRPA infection patients, especially those whose SOFA score is ≥ 2 or whose Charlson comorbidity index is ≥ 6.

## Introduction

*Pseudomonas aeruginosa* (*P. aeruginosa*) is a ubiquitous microorganism that causes different types of infections, primarily in immunosuppressed patients, critical care patients, or those with comorbidities [1]. *P. aeruginosa* is also one of the most common pathogens that causes nosocomial infections, including central line-associated bloodstream infections, ventilation-associated pneumonia [1].

For clinicians, selecting an appropriate antibiotic regimen for Carbapenem-resistant *P. aeruginosa* (CRPA) infections is a global challenge. Instead of carbapenems, other antibiotics, such as ceftolozane-tazobactam, aminoglycoside, and fosfomycin, can be used against CRPA [2, 3]. The rationale for combination anti-infective therapy against CRPA is based on the possibility of achieving a higher rate of bacterial killing. Current clinical guidelines suggest using combination therapy to treat patients with severe CRPA infections [2, 3]. However, it is a conditional recommendation for use, and insufficient evidence is available for this recommendation. Current guidelines do not provide a specific method to identify severe CRPA infections [2, 3]. Additionally, recent studies have demonstrated that the effects of monotherapy and combination therapy on the prognosis of CRPA infection remain controversial [4–7].

Furthermore, there is growing evidence of harm caused to individual patients by unnecessary antimicrobial use. More antibiotic use risks higher costs, and it is more likely to cause drug-related adverse events such as allergic or hypersensitivity reactions and kidney injury [8]. In addition to adverse drug reactions and drug toxicity, antimicrobial resistance is one of the most widely recognized mechanisms of antimicrobial-associated harm [8]. A prospective observational study found that concomitant use of several antimicrobials is associated with an excess mortality risk compared to monotherapy [9].

Sepsis is characterized by fatal organ dysfunction caused by an overwhelming host response to an infection. The sequential organ failure assessment (SOFA) score is predominantly used to assess the severity of organ dysfunction and the severity of septic shock in sepsis [10, 11]. The primary objective of this study is to determine the effectiveness of anti-Infective combination therapy compared to monotherapy in achieving clinical success in patients with CRPA infection and risk factors of clinical failure of monotherapy.

## Methods

### Study design

We conducted a retrospective cohort study from Medical Information Mart for Intensive Care IV (MIMIC-IV). MIMIC-IV is a database that included more than 2 million anonymized patients who were admitted to the critical care units of Beth Israel Deaconess Medical Center (BIDMC) from 2008 to 2019.

### Database

This study utilized data from MIMIC-IV. Clinical variables such as demographics, comorbid diseases, laboratory tests, microbial test results, and antibiotic records were documented in this database. The study authors have already completed the training course to gain access to the database.

### Study population

The inclusion criteria were (1) age ≥18 years at admission and (2) infection caused by CRPA. The exclusion criteria were as follows: 1) length of stay shorter than 3 days, 2) missing information about CRPA, and 3) no antibiotic against CRPA or duration of antibiotics against CRPA shorter than 3 days. To avoid the potential impact of previous antibiotic use, we also excluded patients who had received monotherapy with different types of antibiotics.

### Data extraction

The Structured Query Language (SQL) with the PostgreSQL tool (version 9.6) was used to extract data from MIMIC-IV. The extracted data included demographics, SOFA scores, complete antibiotic records 10 days after CRPA was found, and laboratory tests. We also extracted the comorbidities, including hypertension, diabetes, myocardial infarction, chronic pulmonary disease, renal failure, cerebrovascular disease, and severe liver disease.

### Definition

Clinical success events were as follows: 1). Correction of septic shock (systolic blood pressure >90 mm Hg without the need for vasopressor support); 2). Ventilation withdrawal for patients with pneumonia; 3), microbiological cure for patients with bacteremia (no growth in the blood of an index isolate on day 14 or before); and 4) improved or stable SOFA score (for baseline SOFA ≥3, we required that the score improve by at least 30%, and for a baseline SOFA < 3, we required that the score remain the same or decrease). Clinical failure events were as follows: 1). new septic shock (systolic blood pressure <90 mm Hg with vasopressor therapy); 2) new ventilation for patients with pneumonia; 3) new bacteremia; and 4) increased SOFA score (for SOFA ≥3, we required that the score increase more than 30%, and for baseline SOFA < 3, we required that the score be greater than 3).

Similar to a previous study [12], clinical success was defined as a composite of patient survival, at least one clinical success event, and no clinical failure events 14 days after CRPA was found. Complete clinical success was defined as a composite of patient survival and all clinical success events 14 after days CRPA was found. Patients who did not meet the clinical success criteria were classified as having clinical failure. The outcomes of this study were clinical success, complete clinical success, and 28-day all-cause mortality.

### Statistical analysis

Continuous variables are described as the means and standard deviations and were compared pairwise with Student's t test and one-way ANOVA across groups. Categorical variables are presented as numbers and percentages and were compared using Pearson’s chi-square test or Fisher’s exact test as appropriate. We performed a survival analysis using the log-rank test and 28-day Kaplan-Meier curves. Univariate and multivariate analyses for assessing independent risk predictors were performed using the logistic regression model. All statistical analyses were performed using SPSS software (v23.0; IBM, Armonk, NY); a two-sided *P*<0.05 was considered statistically significant.

## Results

### Characteristics of different antibiotic regimens and outcomes patients

**Fig. 1:**
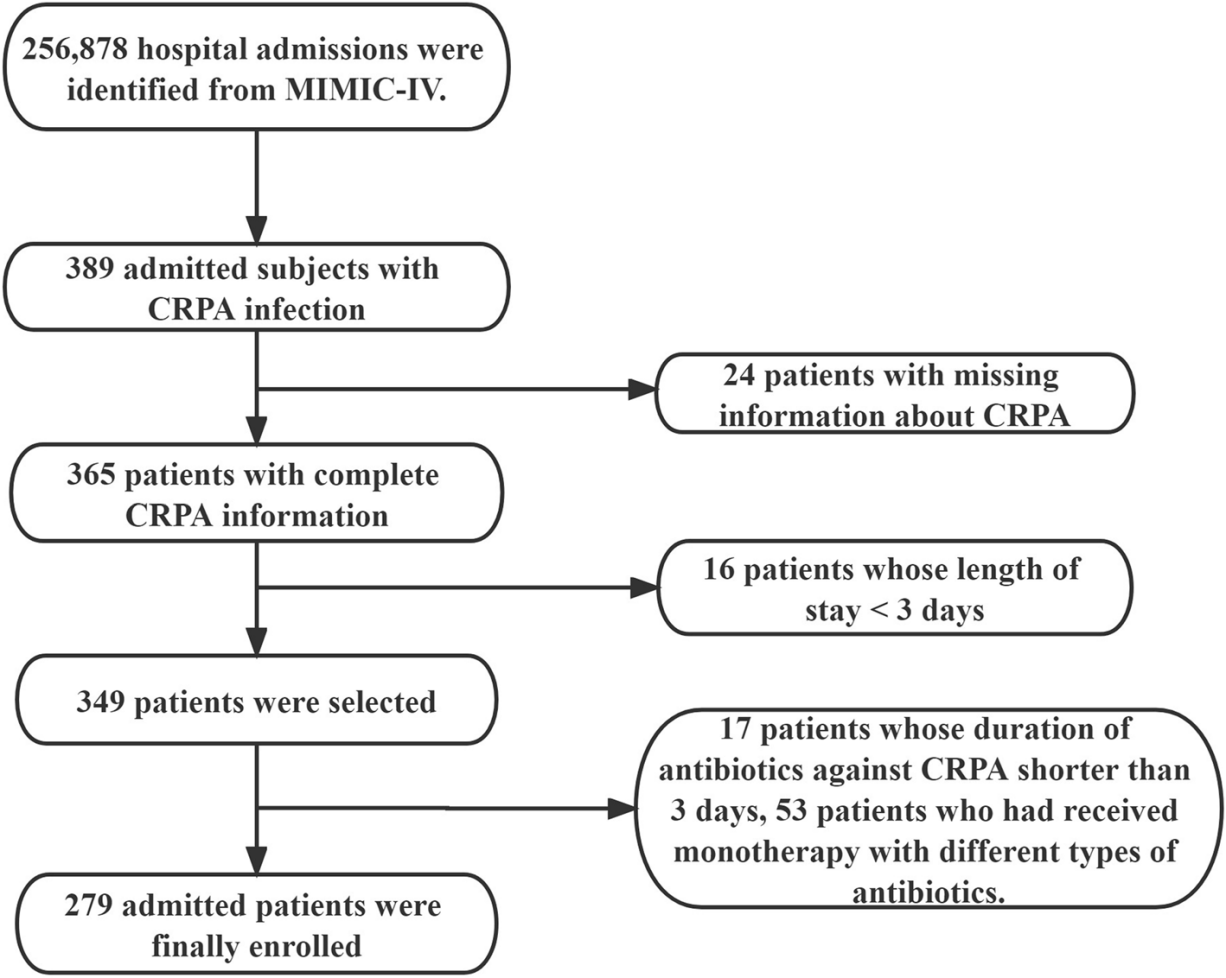
Flow diagram for patient recruitment from the Multiparameter Intelligent Monitoring in Intensive Care IV database

As shown in Fig. 1 (Title: Flow diagram for patient recruitment from the Multiparameter Intelligent Monitoring in Intensive Care IV database, Legends: This figure presented the patient recruitment process. Inclusion criteria in this study: 1. Age ≥ 18 years at admission. 2. Infection caused by Carbapenem-Resistant Pseudomonas aeruginosa (CRPA). Exclusion criteria in this study: 1. Length of stay shorter than 3 days. 2. Missing information about CRPA. 3. No antibiotic treatment against CRPA or duration of antibiotics against CRPA shorter than 3 days.4. Patients who received monotherapy with different types of antibiotics were also excluded to avoid potential impact of previous antibiotic use. Final sample: 279 patients meeting all inclusion criteria and not falling into any of the exclusion criteria were included in the final study sample.), a total of 279 subjects were enrolled for our final data analysis after screening by the inclusion and exclusion criteria. We excluded 110 patients based on various excluded criteria above.

Table 1 shows the characteristics of the monotherapy and combination therapy patients. Most of the patients in our study were white men, at approximately 70.25%. Regarding ethnicity, sex, and age, we found no difference between the two groups. The proportion of diabetic patients in patients with monotherapy was higher than that in patients with combination therapy (49.5% versus 33.9%, *p*= 0.012). The proportions of other comorbid diseases between the 2 groups were similar. Therefore, monotherapy and combination therapy patients have similar Charlson comorbidity indices. Our study ensured the accuracy of microorganism culture results in identifying infections rather than colonization through a comprehensive approach. This included considering clinical diagnosis by physicians, reviewing the clinical histories of infected patients, and conducting a meticulous review of medical records in the MIMIC-IV database to confirm the presence of infections. We included adults with infections caused by CRPA, primarily bacteremia, pneumonia, urinary tract infections, and tissue infections. Sputum was the most common specimen source, followed by urine. In this study, 27 (29.0%) patients receiving monotherapy and 70 (37.6%) patients receiving combination therapy also received ventilation support. Fifty patients with CRPA infections developed septic shock. The rate of clinical success for combination therapy was higher than that of monotherapy (73.1% versus 60.4%, *p*=0.028). We did not find a significant difference in complete clinical success between the 2 groups (36.2% versus 42.0%, *p*=0.931).

**Table 1:** Characteristics of Patients with Carbapenem-resistant Pseudomonas aeruginosa (CRPA) infections according to antibiotic treatments (monotherapy vs combination regimen)

	Overall(*N*=279)	Monotherapy(*N*=93)	Combination therapy(*N*=186)	*p* value
**Age(year)**	66(56,75)	67(56,75)	65(56,75)	0.597
**Male,n(%)**	180(64.5%)	59 (63.4%)	121 (65.1%)	0.791
**Ethnicity,n(%)**				0.616
White	196(70.3%)	63(67.7%)	133(71.5%)	
Black/African American	29(10.4%)	12(12.9%)	17(9.1%)	
Others	54(19.4%)	18(19.4%)	36(19.4%)	
**Comorbidity,n(%)**				
Hypertension	68(24.4%)	21(22.6%)	47(25.3%)	0.622
Diabetes	109(39.1%)	46(49.5%)	63(33.9%)	0.012
Myocardial infarction	42(15.1%)	16(17.2%)	26(14.0%)	0.478
Chronic pulmonary disease	90(32.3%)	25(26.9%)	65(34.9%)	0.174
Cerebrovascular disease	36(12.9%)	15(16.1%)	21(11.3%)	0.256
Renal disease	83(29.7%)	29(31.2%)	54(29.0%)	0.688
Severe liver disease	13(4.7%)	5(5.4%)	8(4.3%)	0.155
**Laboratory test**				
White cell count(*10^9^/l)	10.77(7.80,15.20)	10.50(7.93,14.60)	10.81(7.50,15.70)	0.675
Hemoglobin( g/dl)	8.70(7.97,9.70)	8.70(7.93,9.90)	8.73(7.97,9.70)	0.903
Platelet (*10^9^/l)	221.67(144.33,328.00)	249.75(155.00,345.00)	218.67(134.57,322.33)	0.417
Creatinine(mg/dl)	0.90(0.60,1.53)	1.00(0.70,1.60)	0.90(0.60,1.53)	0.358
**Site of infection,n(%)**				0.273
Respiratory tract	138(49.5%)	42(45.2%)	96(51.6%)	
Urinary tract	54(19.4%)	20(21.5%)	34(18.3%)	
Bloodstream	24(8.6%)	5(5.4%)	19(10.2%)	
Other	63(22.6%)	26(28.0%)	37(19.9%)	
**Ventilation,n(%)**	97(34.8%)	27(29.0%)	70(37.6%)	0.155
**Septic shock,n(%)**	50(17.9%)	11(11.8%)	39(21.0%)	0.061
**Charlson Comorbidity Score**	6(4,8)	6(5,8)	6(4,8)	0.770
**SOFA score**	4(1,7)	3(1,6)	4(1,7)	0.312
**Outcome,n(%)**				
Clinical success	192(68.8%)	56(60.2%)	136(73.1%)	0.028
Complete clinical success	109(39.1%)	51(36.2%)	58(42.0%)	0.931
28-day mortality	49(17.6%)	14(15.1%)	35(18.8%)	0.436

We also compared different characteristics of clinical failure patients with those of clinical success patients (Table 2). Regarding ethnicity, sex, and age, we found no difference between the two groups. The proportion of renal disease patients in the clinical failure group was higher than that in the clinical success group (37.9% versus 26.0%, *p*= 0.044). Compared to patients in the clinical failure group, patients in the clinical success group were more likely to die within 28 days after CRPA was found (48.3% versus 3.6%, *p*<0.001).

**Table 2:** Characteristics between clinical success and clinical failure patients

	Clinical failure(*N*=87)	Clinical success (*N*=192)	*p* value
**Monotherapy**	37(42.5%)	56(29.2%)	0.028
**Combination therapy**	50(57.5%)	136(70.8%)	0.028
**Age(year)**	64(57,75)	66(55,75)	0.658
**Male,n(%)**	56(64.4%)	124(64.6%)	0.972
**Ethnicity,n(%)**			0.013
White	56(64.4%)	140(72.9%)	
Black/African American	16(18.4%)	13(6.8%)	
Others	15(17.2%)	39(20.3%)	
**Comorbidity,n(%)**			
Hypertension	18(20.7%)	50(26.0%)	0.335
Diabetes	38(43.7%)	71(37.0%)	0.288
Myocardial infarction	13(14.9%)	29(15.1%)	0.972
Cerebrovascular disease	14(16.1%)	22(11.5%)	0.285
Chronic pulmonary disease	27(31.0%)	63(32.8%)	0.769
Renal disease	33(37.9%)	50(26.0%)	0.044
Severe liver disease	3(3.4%)	10(5.2%)	0.518
**Laboratory test**			
White cell count(*10^9^/l)	10.77(8.00,15.10)	10.75(7.50,15.24)	0.766
Hemoglobin( g/dl)	8.80(7.97,9.90)	8.70(7.95,9.70)	0.737
Platelet(*10^9^/l)	196.00(143.00,279.00)	237.50(146.00,315.50)	0.067
Creatinine(mg/dl)	1.00(0.60,2.40)	0.90(0.60,1.42)	0.079
**Site of infection,n(%)**			0.247
Respiratory tract	47(54.0%)	91(47.4%)	
Urinary tract	18(20.7%)	36(18.8%)	
Bloodstream	4(4.6%)	20(10.4%)	
Other	18(20.7%)	45(23.4%)	
**Ventilation,n(%)**	32(36.8%)	65(33.9%)	0.634
**Septic shock,n(%)**	13(14.9%)	37(19.3%)	0.383
**Charlson Comorbidity Score**	6(5,9)	6(4,7)	0.069
**SOFA score**	4(2,6)	3(0,7)	0.244
**Outcome,n(%)**			
28-day mortality	42(48.3%)	7(3.6%)	<0.001

### Kaplan-Meier analysis

**Fig. 2:**
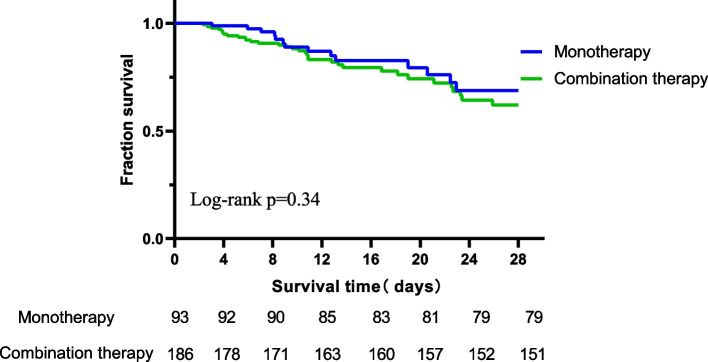
Comparison of All-Cause Mortality within 28 Days After CRPA Detection between Monotherapy and Combination Therapy

In Fig. 2 (Title: Comparison of All-Cause Mortality within 28 Days After CRPA Detection between Monotherapy and Combination Therapy, legend: This figure compares all-cause mortality within 28 days after CRPA detection for Monotherapy and Combination Therapy. X-axis: Time (days) after CRPA detection. Y-axis: Proportion of surviving patients. Blue curve: Monotherapy. Green curve: Combination Therapy. Log-rank test, *p*-value = 0.34, indicates no significant difference. At 28 days, 79 survived in Monotherapy, and 151 in Combination Therapy.), no significant difference was observed between patients who received monotherapy and those who received combination therapy for all-cause mortality within 28 days after CRPA was found (21.3% vs. 13.8%).

### Univariate and multivariate logistic regression models

To verify the effect of monotherapy and combination therapy on clinical efficacy through univariate and multivariate logistic regression analyses, the covariates listed in Table 1 were used. Model 1 is the unadjusted model. Model 2 was adjusted for model 1 plus age ≥60 years, sex, and ethnicity. Model 3 was adjusted for model 2 plus comorbidities: myocardial infarction, chronic pulmonary disease, and renal disease; and model 4 was adjusted for model 3 plus ventilation, septic shock, the SOFA score, and the Charlson comorbidity index (Table 3).

**Table 3:** Multivariate analysis for clinical success and complete clinical success

**Clinical success**				**Complete clinical success**		
Regression Models	OR	95%CI	*p* value	OR	95%CI	*p* value
Model 1	0.581	0.354-0.953	0.032	0.978	0.587-1.629	0.931
Model 2	0.574	0.336-0.981	0.042	0.988	0.590-1.655	0.964
Model 3	0.575	0.334-0.989	0.046	0.942	0.554-1.559	0.824
Model 4	0.559	0.321-0.976	0.041	0.661	0.518-1.517	0.887

Model 1:unadjusted

Model 2 adjusted for model 1 plus age≥60 years, gender, ethnicity

Model 3 adjusted for model 2 plus myocardial infarction, chronic pulmonary disease and renal disease

Model 4 adjusted for model 3 plus ventilation, septic shock, SOFA score, Charlson Comorbidity Index

*OR* odds ratio

After adjusting each model listed above, monotherapy was found to be significantly correlated with clinical success (model 1: odds ratio (OR), 0.581; 95% confidence interval (CI), 0.354-0.953; *p*=0.032; model 2: OR, 0.574; 95% CI, 0.336-0.981; *p* = 0.042; model 3: OR, 0.575; 95% CI, 0.334-0.989; *p* = 0.046; and model 4: OR, 0.559, 95% CI, 0.321-0.976; *p* = 0.041). However, we found that monotherapy was not related to complete clinical success (model 1: OR, 0.978; 95% CI, 0.587-1.629; *p* = 0.931; model 2: OR, 0.988; 95% CI, 0.590-1.655; *p* = 0.964; model 3: OR, 0.942; 95% CI, 0.554-1.559; *p* = 0.824; and model 4: OR, 0.661, 95% CI, 0.518-1.517; *p* = 0.887).

### Subgroup analyses

To rule out the potentially confounding influences of gender, SOFA, and Charlson comorbidity index, subgroup analyses were performed (Table 4). Monotherapy may be a risk factor for clinical failure in male patients (OR:0.441, 95%CI:0.215-0.904) or patients with a SOFA ≥2 or a Charlson comorbidity index≥6 (OR: 0.469, 95% CI: 0.231-0.953; and OR: 0.467, 95% CI: 0.222-0.980). We did not find an association between monotherapy and complete clinical success or 28-day mortality.

**Table 4:** Subgroup analyses of the association between main clinic outcomes and monotherapy stratified by outcome predictors

	Adjusted OR (95% CI)	*p* value	Adjusted OR (95% CI)	*p* value
	Male(*n*=180)		Female(*n*=99)	
Clinical success	0.441(0.215-0.904)	0.025	0.840(0.327-2.161)	0.717
Complete clinical success	1.219(0.615-2.415)	0.571	0.599(0.233-1.540)	0.287
28-day mortality	1.000(0.422-2.371)	1.000	0.557(0.30-2.383)	0.430
	SOFA < 2(*n*=96)		SOFA ≥ 2(*n*=183)	
Clinical success	0.599(0.190-1.893)	0.383	0.469(0.231-0.953)	0.036
Complete clinical success	1.065(0.429-2.642)	0.891	0.697(0.332-1.463)	0.340
28-day mortality	0.777(0.191-3.163)	0.724	0.906(0.376-2.185)	0.826
	Charlson Comorbidity Score < 6(*n*=118)		Charlson Comorbidity Score ≥ 6(*n*=161)	
Clinical success	0.626(0.250-1.565)	0.316	0.467(0.222-0.980)	0.044
Complete clinical success	0.715(0.309-1.656)	0.434	1.037(0.502-2.142)	0.922
28-day mortality	2.089(0.596-7.322)	0.250	0.546(0.208-1.433)	0.219

## Discussion

The primary objective of this study is to determine the effectiveness of anti-Infective combination therapy compared to monotherapy in achieving clinical success in patients with CRPA infection and risk factors of clinical failure of monotherapy. In this study, we found that combination therapy is recommended for CRPA infection patients, especially those whose SOFA score was ≥2 or whose Charlson comorbidity index was ≥6. Regarding all-cause 28-day mortality, we did not find any differences between the monotherapy and combination therapy groups.

CRPA has emerged and caused many nosocomial outbreaks, leading to millions of deaths each year [13, 14]. As the carbapenem-resistance mechanisms are complicated, selecting an effective and safe antibiotic regimen remains a challenge. Traditional antipseudomonal β-lactams, including cefepime, ceftazidime and piperacillin/tazobactam and new BLBLIs (ceftazidime-avibactam and ceftolozane-tazobactam), have been found to be effective for CRPA [2, 3]. To increase the success rates of treatment, researchers have attempted to identify the most effective antibiotic regimens against CRPA. In this study, we also found that combination therapy was more effective than monotherapy in patients with CRPA infection. However, the effectiveness of combination therapy for CRPA infections remains controversial. One way of assessing the effectiveness of antibiotic regimens involves in vitro methods based on bacterial killing and antibiotic synergism [15, 16]. According to Ramos JF [4] et al., meropenem with colistin demonstrated superior performance compared to other antibiotic regimens. In vivo CRPA models were also created to test the efficacy of antibiotic regimens. In an intraperitoneal murine CRPA infection model, combination therapy involving colistin and rifampicin exerted a synergistic effect [17]. A previous study demonstrated that the combination of caspofungin and polymyxin exhibited superior efficacy compared to caspofungin alone in reducing mixed biofilm biomass and fungal and bacterial viability in CRPA strains [18]. Similarly, the combination of ceftazidime/avibactam (CZA) with aztreonam (ATM) showed synergistic bacteriostatic or bactericidal activity against NDM-, IMP-, KPC+IMP-, and KPC+NDM-producing carbapenem-resistant Enterobacterales (CRE) strains. This combination therapy not only reduced mortality but also prolonged the lifespan of mice infected with these strains [19]. Furthermore, a combination therapy involving aggressive doses of polymyxin B and tigecycline displayed synergistic or additive effects in treating multidrug-resistant carbapenem-resistant *Acinetobacter baumannii*(CRAB) infections in humans [20]. In a previous retrospective cohort study included critically ill patients with CRAB infections, they found that patients who received the combination therapy of colistin plus meropenem had a significantly lower adjusted odds ratio (aOR) for 30-day mortality compared to those who received colistin monotherapy. The aOR for 30-day mortality was 0.43, with a 95% confidence interval (CI) ranging from 0.23 to 0.82 [21]. The results of previous study showed that CRAB infections patients who received loading dose (LD) colistin-imipenem had a lower 30-day survival rate (adjusted HR = 0.57, 95% CI: 0.37-0.90; *p* = 0.015) and a lower clinical response (aHR = 0.56, 95% CI: 0.35-0.90; *p* = 0.017) compared with those who received LD colistin-meropenem [22].

However, combination therapy did not improve clinical outcomes in all patients with carbapenem-resistant bacteria infection. In a study by Mical Paul [12] et al., which included 406 patients, no significant difference was observed in clinical failure at 14 days after randomization between the colistin monotherapy group (156/198, 79%) and the combination therapy group (152/208, 73%) (risk ratio [RR] 0.93, 95% CI 0.83-1.03). Regarding infections caused by CRAB, the combination of colistin with vancomycin did not show any significant differences in 30-day mortality, clinical response, or microbiological response compared to colistin alone. The rates of nephrotoxicity were similar in both groups, suggesting that colistin combination therapy with vancomycin may not be necessary for managing CRAB infections [23]. A recent meta-analysis that involved 11 studies and 396 patients with carbapenem-resistant gram-negative bacteria (CRGNB) receiving ceftazidime/avibactam alone or in combination was conducted. There was no significant difference in the mortality rate and microbiological cure rate between combination therapy and monotherapy (38.1% vs. 30.9%; 64.9% vs. 63.4%), according to the meta-analysis [24]. In Western China, a retrospective study of 355 patients with Carbapenem-resistant Gram-negative bacterial bloodstream infections (CRGNB-BSI) found that Combination antimicrobial therapy was not superior to monotherapy (*P* = 0.387) and appropriate therapy was associated with lower treatment failure and 28-day in-hospital mortality rates [25]. Another retrospective study of 164 CRE bloodstream infection cases in China, highlighted the importance of early detection of carbapenemase type and timely initiation of appropriate combination therapy for improving survival [26]. A randomized clinical trial investigated the association between mortality in Acinetobacter baumannii infections and colistin resistance, showed that colistin monotherapy yielded better outcomes compared to colistin-meropenem combination therapy for patients with colistin-resistant isolates [27]. Current guidelines do not take a definitive stance on whether to recommend or discourage the use of combination therapy with new antibiotics (ceftazidime-avibactam and ceftolozane-tazobactam) or cefiderocol for CRPA infections [2, 3]. Overall, these studies underscore the importance of exploring combination therapies to combat carbapenem-resistant bacterial infections. While some combinations show promising results and improved outcomes, it's essential to recognize that not all combination therapies may be effective in every case. The severity of the infection, the specific bacterial strain, and individual patient factors may influence the success of combination therapies. Further research and clinical trials are necessary to identify the most effective treatment strategies for combating carbapenem-resistant bacteria infections.

In addition to finding the most effective antibiotic regimens, identifying the patients who benefit most from monotherapy is also important. Antibiotics can harm patients by various mechanisms: drug toxicity, mitochondrial dysfunction and organ dysfunction, adverse drug reactions, and antimicrobial resistance [8]. Therefore, in order to prevent harm caused by antimicrobials, doctors should limit their use to only when necessary. For patients with non-severe CRPA infections, current guidelines also recommend individualized selection of in vitro-active monotherapy based on the source and type of infection. However, current guidelines do not provide specific tools to evaluate the severity of CRPA infections [2, 3]. SOFA is one of the most frequently used tools to screen and evaluate the severity of sepsis and is also shown to be closely related to the outcomes of sepsis patients [28]. The SOFA score is required for accurate assessment [29]. Combination therapy consisting of two antimicrobials with gram-negative coverage for empiric treatment is recommended for patients with sepsis or septic shock and a high risk for multidrug-resistant (MDR) organisms [30]. In our study, we also found that combination therapy is beneficial for CRPA-infected patients whose SOFA score was ≥ 2 points to control infection. Similarly, SOFA is recommended to help physicians evaluate clinical CRE infection severity before meropenem-based combination therapy is performed [31].

In addition to organ dysfunction, disease burden should be taken into consideration when physicians evaluate the outcomes of infection. The Charlson comorbidity index has been a useful, simple, and readily applicable tool for physicians in their effort to assess and underline complicated diseases and an indicator of disease burden. Its weighted score was assigned to 17 comorbidities and ages, which were found to be associated with long-term mortality [32]. Patients with weakened immune systems or existing health conditions are more vulnerable to *P. aeruginosa* and are at greater risk of developing severe infections like septic shock or sepsis [33–35]. The presence of comorbidities is a crucial factor that affects the prognosis of respiratory system infections. Comorbidities such as cardiovascular disease and cancer, which are included in the Charlson comorbidity index, were associated with worse outcomes in cases of acute respiratory infections [36, 37]. The administration of appropriate antimicrobials is one of the most important interventions to control the infection.

In this study, we also explored which antimicrobial regimen (monotherapy or combination therapy) was beneficial to achieving clinical anti-infection treatment success for those patients who had more comorbid diseases. According to the subgroup analysis results, we further found that combination therapy is more suitable for CRPA-infected patients whose Charlson comorbidity index is not less than 6 to control infection.

Our study has the following limitations. First, it is a retrospective study from a large database, and further random control trials are needed. Second, the data was obtained from the MIMIC-IV database,which may limit the generalizability of the findings to other settings. Different hospitals or regions may have variations in patient populations, treatment protocols, and antimicrobial resistance patterns, which could affect the outcomes. Third, the study included a relatively small sample size (279 subjects), which may limit the statistical power and generalizability of the findings. A larger sample size would provide more robust results and allow for more detailed subgroup analyses. Forth, there are some *P. aeruginosa* antibiotic susceptibilities results that do not include β-lactam agent, so we do not know if β-lactams are effective against these *P. aeruginosa* bacteria in vitro.Fifth, potential unmeasured confounders: Despite adjusting for various factors in the multivariate analysis, there may still be unmeasured confounding variables that were not accounted for. These variables could potentially influence the outcomes and introduce bias into the results. Sixth,we had no access to the specific carbapenem-resistance mechanisms of CRPA isolated from patients in our study. Seventh, we did not find the most effective combination therapy regimens for CRPA due to the limitation in the original data.

## Conclusion

Combination therapy is more effective for CRPA infection patients, especially those whose SOFA score is ≥ 2 or whose Charlson comorbidity index is ≥ 6. SOFA and Charlson comorbidity index may help clinicians to decide antibiotic regimen and to avoid using unnecessary antibiotics.

## Data Availability

Anyone should be obliged to complete the training course to gain access to the database. Data associated with this study has been deposited. The data are available on the MIMIC-IV database website at https://mimic-iv.mit.edu/.
